# HFE gene variants, iron, and lipids: a novel connection in Alzheimer’s disease

**DOI:** 10.3389/fphar.2014.00165

**Published:** 2014-07-08

**Authors:** Fatima Ali-Rahmani, Cara-Lynne Schengrund, James R. Connor

**Affiliations:** ^1^Departments of Neurosurgery, Neural and Behavioral Sciences and Pediatrics, Center for Aging and Neurodegenerative Diseases, Penn State Hershey Medical CenterHershey, PA, USA; ^2^Departments of Biochemistry and Molecular Biology, The Pennsylvania State University College of MedicineHershey, PA, USA

**Keywords:** iron, cholesterol, sphingolipids, Alzheimer disease, HFE, H63D, brain

## Abstract

Iron accumulation and associated oxidative stress in the brain have been consistently found in several neurodegenerative diseases. Multiple genetic studies have been undertaken to try to identify a cause of neurodegenerative diseases but direct connections have been rare. In the iron field, variants in the HFE gene that give rise to a protein involved in cellular iron regulation, are associated with iron accumulation in multiple organs including the brain. There is also substantial epidemiological, genetic, and molecular evidence of disruption of cholesterol homeostasis in several neurodegenerative diseases, in particular Alzheimer’s disease (AD). Despite the efforts that have been made to identify factors that can trigger the pathological events associated with neurodegenerative diseases they remain mostly unknown. Because molecular phenotypes such as oxidative stress, synaptic failure, neuronal loss, and cognitive decline, characteristics associated with AD, have been shown to result from disruption of a number of pathways, one can easily argue that the phenotype seen may not arise from a linear sequence of events. Therefore, a multi-targeted approach is needed to understand a complex disorder like AD. This can be achieved only when knowledge about interactions between the different pathways and the potential influence of environmental factors on them becomes available. Toward this end, this review discusses what is known about the roles and interactions of iron and cholesterol in neurodegenerative diseases. It highlights the effects of gene variants of HFE (H63D- and C282Y-HFE) on iron and cholesterol metabolism and how they may contribute to understanding the etiology of complex neurodegenerative diseases.

## INTRODUCTION

The average increase in life expectancy has been accompanied by an increase in the number of people with dementia, a problem expected to affect half of those living to be 85 or older. The most prevalent form of dementia is Alzheimer’s disease (AD) and its incidence is expected to triple by 2050 (Hebert et al., 2013). The observations that more than 40% of AD patients carried the ApoE4 allele (Cedazo-Minguez and Cowburn, 2001) and that those carrying both the ApoE4 allele and expressing the H63D variant of the hemochromatosis protein HFE were prone to earlier onset of AD (Moalem et al., 2000; Percy et al., 2008) support the hypothesis that disruption of the normal metabolism of both iron and cholesterol contribute to AD. More specifically, the need for a discussion of the role of iron and cholesterol in neurodegenerative disease stems from our observation that disruption of normal iron metabolism in H63D-HFE-expressing human neuroblastoma cells resulted in altered cholesterol metabolism as well as our findings that mice expressing the orthologous H67D-HFE had alterations in brain iron and cholesterol metabolism and a reduction in brain volume that correlated with poorer recognition and spatial memory, symptoms associated with AD (Ali-Rahmani et al., 2014a).

## IRON IN NEURODEGENERATIVE DISEASES

Numerous studies have implicated metals such as iron, copper, zinc, and aluminum in the pathogenesis of AD (Sayre et al., 2000; Connor et al., 2001; Bush, 2003; Maynard et al., 2005; Connor and Lee, 2006; Liu et al., 2006; Roberts et al., 2012). Evidence indicates that oxidative stress induced by excess iron contributes to neurodegeneration (Castellani et al., 2007). Because iron is the most abundant transition metal in the body and is readily available from several dietary options, it is important to understand how its regulation in the body is influenced by other factors and how its elevation or depletion affects cellular processes that could lead to AD pathogenesis. To understand the role of iron in neurodegeneration, it is necessary to understand its normal function and regulation.

### ROLE OF IRON IN BRAIN

Iron is essential for a number of cellular processes needed for survival. It is a required cofactor for a number of enzymes involved in cell functions such as energy production (mitochondrial electron transport chain), DNA synthesis and repair, ribosome biogenesis, neurotransmitter synthesis, myelin synthesis and lipid metabolism, and cell cycle regulation. Iron is also needed for heme production and formation of iron-sulfur clusters that are essential for electron transport and DNA repair (Rouault and Tong, 2005; Tong and Rouault, 2006). Though these reactions occur in every organ, the role of iron in the brain is particularly important. Brain has the highest demand for oxygen of all organs and iron-containing neuroglobin is essential to meet this need. In addition, to maintain the complex communication network and to establish plasticity, the brain constantly remodels cell contacts and synapses. These processes rely on protein synthesis by ribosomes which, in turn, depend on [4Fe–4S] cluster-containing proteins (Kispal et al., 2005). The iron-sulfur clusters are also essential for DNA synthesis (Netz et al., 2012). Moreover, iron is an essential trophic factor needed for myelination. This is because key enzymes such as 3-hydroxy-3-methylglutaryl-coenzyme A reductase (HMGCoAR), squalene synthase, and glucose-6-phosphate dehydrogenase needed for the biosynthesis of myelin cholesterol and lipids require iron as a cofactor (Lange and Que, 1998; Todorich and Connor, 2004). The role of iron in these processes is thought to reflect its ability to (1) donate electrons needed for redox reactions, (2) transfer electrons in mitochondria, and (3) to bind oxygen in heme. But if iron accumulates in an unchelated form, its ability to readily exchange electrons can result in formation of reactive free radicals by the Fenton reaction (Lloyd et al., 1997). In turn, this can lead to oxidative stress that can negatively impact a number of cellular processes and disrupt cell membrane integrity. Because oxidative stress is considered a causative agent in the etiology of AD (Castellani et al., 2007; Cai et al., 2011), the role of iron and disorders resulting in iron overload have been under intense investigation for their potential role as a risk factor for AD.

### IRON IN AD

Several studies have reported that brain iron content and expression of iron-regulating proteins such as ferritin (Frt; high expression indicates more iron) increase with age, but the increase in iron is greater than ferritin thus suggesting the increased iron is not stored properly (Hallgren and Sourander, 1958; Milton et al., 1991; Connor et al., 1992a,b, 1995; Bartzokis et al., 1994, 2007; Martin et al., 1998; Hirose et al., 2003). The finding that iron accumulation is significantly higher in brains of patients with AD or mild cognitive impairment (MCI) than in those of age-matched non-demented controls (Ding et al., 2009) supports the proposal that the increase in iron underlies age-related cognitive decline (Bartzokis, 2009). Disturbances in iron metabolism have been found in post-mortem brain tissue from patients with AD, in cerebrospinal fluid (CSF) and *in vivo* using magnetic resonance imaging (MRI). Regions where iron deposition was found include the hippocampus, basal ganglia and cortex (Loeﬄer et al., 1995; Bartzokis et al., 2000; Ding et al., 2009) as well as senile plaques and neurofibrillary tangles and cells surrounding them (Connor et al., 1992a). Moreover, higher brain iron content was also found to correlate with the severity of cognitive impairment (Zhu et al., 2009). Thus, use of MRI to monitor brain iron has been proposed as a method for assessing disease progression. Excess unchelated iron is considered a major cause of oxidative stress that can lead to modification of proteins, lipids, DNA, and RNA, thereby, inducing several features associated with AD (Markesbery, 1997; Ferrari, 2000). These alterations are toxic to cells because they result in activation of cell apoptosis pathways and eventually cell death. In fact, markers of protein oxidation and lipid peroxidation were consistently found to be elevated in the brains and CSF samples from AD patients and those with MCI who subsequently developed AD (Markesbery, 1997; Pratico et al., 2002; Brys et al., 2009). Moreover, markers of lipid peroxidation in ventricular fluid were reported to correlate with cortical atrophy, reduced brain weight and severity of AD (Montine et al., 1999). Proteins involved in maintaining iron homeostasis include the transferrin receptor (TfR; iron uptake), transferrin (Tf; iron transport), ferritin (Frt; iron storage), HFE (iron uptake), ceruloplasmin (iron transport; feroxidase- conversion of Fe^2+^ to Fe^3+^), DMT1 (iron export), and ferroportin (iron export). Indeed, alterations in the expression pattern of Tf, Frt, and HFE were found in brains of AD patients and are discussed below. Moreover, genetic mutations in *Tf* (C2 variant; rs1049296) and two variants of *HFE,* C282Y (rs1800562) and H63D (rs1799945), were shown to affect body iron status (Fleming et al., 2000; Bartzokis et al., 2010; Kauwe et al., 2010), and oxidative stress (Loeﬄer et al., 1995; Lleo et al., 2002). Where H63D has been associated with increased risk of AD, C282Y has been found to be protective for AD. There is substantial evidence for the role of iron overload in AD that is further supported by epidemiological evidence implicating variants of iron management proteins in AD risk. The AlzGene meta-analysis of the Tf C2 allele (Bertram et al., 2007; www.alzgene.org/) currently shows a significant, although low, odds ratio of AD: 1.2 (95% confidence interval, 1.06–1.3; June 2010), with a similar pattern in Caucasians and east Asians.

### IRON, HFE, AND AD

Iron accumulation in the brain is accompanied by an increase in oxidative stress that is consistently observed in AD (Connor et al., 1992a; Markesbery, 1997; Liu et al., 2005; Maynard et al., 2005). The high iron (*HFE*) gene product primarily regulates iron uptake into cells by interacting with the TfR to restrict Tf binding (Feder et al., 1998). Genetic variants of the *HFE* gene are unable to maintain iron homeostasis, and in particular, the H63D variant has been under investigation as a potential risk factor for neurodegenerative diseases. Several studies have reported an increased frequency of the H63D mutation in AD patients (Moalem et al., 2000; Sampietro et al., 2001; Combarros et al., 2003; Pulliam et al., 2003; Connor and Lee, 2006). Pulliam et al. (2003) found increased levels of markers of oxidative stress in individuals with HFE mutations compared to controls. The H63D mutation was expressed ∼5 times more frequently in AD patients younger than 70 years compared to patients over 80 (Sampietro et al., 2001). Indeed, MRI studies revealed increased accumulation of iron in carriers of HFE mutations (Nielsen et al., 1995; Berg et al., 2000). Studies have shown that iron staining is most dense in the proximity of amyloid beta (Aβ) plaques and in cells associated with plaques (Connor et al., 1992b) and that iron promotes deposition of Aβ (Rottkamp et al., 2001). Interestingly, a similar pattern was observed with respect to HFE expression in AD brains, suggesting HFE expression may be increased in cells in the vicinity of the plaque. A normal response of HFE is to limit iron uptake thus these cells may be trying to limit iron uptake; a function that would be compromised if the mutant protein is expressed (Connor and Lee, 2006).

### HFE GENE AND VARIANTS

*HFE* (high iron), originally called HLA-H, is a major histocompatibility complex (MHC) class I-like gene. It is located on the short arm of chromosome 6 and was first identified by Simon et al. (1976) in association with an iron overload disorder called hereditary hemochromatosis (HH). The HFE gene encodes a 49 kDa membrane glycoprotein that resembles the MHC-class1 family of proteins. The three most common single nucleotide polymorphisms (SNPs) in the HFE gene are H63D (*rs1799945* in exon 2), C282Y* (rs1800562* in exon 4), and S65C (Merryweather-Clarke et al., 2000; Le Gac et al., 2001). Examination of the frequency and global distribution of HFE mutations indicated that HFE variants are most common in Caucasian populations with a frequency of up to 25% for H63D, 12% for C282Y, and 1.6–5.5% for S65C. S65C is not as common as the other two variants (Camaschella et al., 2002) and has not been associated with physiological changes or diseases (Le Gac et al., 2001).

Not only are the H63D and C282Y variants prevalent, epidemiological evidence associates them with several neurodegenerative, metabolic diseases, and malignancies. Though C282Y is less prevalent in the general population than H63D, it is strongly associated with HH and recently was identified as a risk factor for prostate, breast, colorectal, and brain tumors (Shaheen et al., 2003; Syrjakoski et al., 2006; Osborne et al., 2010; Gannon et al., 2011). It has also been found to protect against neurodegenerative diseases (Buchanan et al., 2002; Correia et al., 2009). In contrast, the H63D variant was identified as a risk factor for several diseases such as AD (Moalem et al., 2000; Sampietro et al., 2001; Combarros et al., 2003; Pulliam et al., 2003; Percy et al., 2008), amyotrophic lateral sclerosis (ALS; Wang et al., 2004; Goodall et al., 2005), and stroke (Ellervik et al., 2007).

### FUNCTION OF HFE

The best characterized function of HFE is its role in regulation of cellular iron uptake which it mediates by binding to the TfR. Binding affinity of HFE for TfR is comparable to the binding affinity of iron-loaded transferrin (holotransferrin; Feder et al., 1998). When a cell has enough iron, the HFE competitively binds to the TfR at the same site as iron-loaded Tf molecules thereby preventing iron uptake via endocytosis. Formation of the HFE-TfR complex is pH-dependent, with a neutral pH allowing tight binding and an acidic pH inhibiting it (Lebron et al., 1998, 1999). HFE can prevent endocytosis of TfR alone by inducing its phosphorylation. This results in higher expression of cell surface TfR than is found intracellularly (Salter-Cid et al., 2000).

The function of HFE in iron regulation is disrupted by mutations causing formation of the H63D- and C282Y-HFE variants. It has been shown that while WT-HFE bound to TfR allows only one iron bound Tf (Fe-Tf) to bind per TfR and be taken up by cells (Lebron et al., 1998, 1999), H63D-HFE allows uptake of more than one molecule of Fe-Tf because it does not reduce the affinity of Fe-Tf for TfR. Because C282Y-HFE is retained in the trans-golgi complex due to its inability to bind β2M and be transferred to the cell surface, it does not interact with TfR, thus resulting in more iron uptake than WT-HFE (Feder et al., 1997; Waheed et al., 1999, 2002). In sum, both variants are associated with cell and tissue iron-overload. For more details see Connor and Lee (2006).

In addition to the increased iron accumulation seen in cells expressing H63D-HFE (Lee et al., 2007; Mitchell et al., 2009b), disruption of mitochondrial potential (Lee et al., 2007), increased influx of intracellular Ca^2+^ (Mitchell et al., 2009b), increased glutamate uptake (Mitchell et al., 2009b), increased secretion of monocyte chemoattractant protein-1 (MCP1) that has a role in neuroinflammation (Mitchell et al., 2009a), increased ER stress (Liu et al., 2011), increased oxidative stress (Lee et al., 2007), increased toxicity to Aβ (Mairuae et al., 2010), and decreased Pin1 activity (Hall et al., 2010) that contributes to increased Tau phosphorylation (Hall et al., 2011) are also found. These findings demonstrate that the expression of H63D-HFE creates a permissive milieu for processes that can influence other pathways in neuronal cells such as lipid homeostasis, neurotransmission, and myelination that may ultimately lead to AD.

## LIPID DYSHOMEOSTASIS IN NEURODEGENERATIVE DISEASES

### CHOLESTEROL METABOLISM IN THE BRAIN

Cholesterol is an essential component of all cell membranes and is needed for maintaining their structure and fluidity. It is required for growth and replication of mammalian cells, exo- and endocytosis, and is a precursor of steroid hormones and bile acids. Cholesterol is synthesized in the liver through a series of reactions with the rate-limiting step catalyzed by HMGCoAR which catalyzes conversion of HMG-CoA to mevalonic acid. Cholesterol is transported to organs other than the brain in the form of lipoproteins. In the brain, almost all of its cholesterol is synthesized *in situ*, with little to none taken up from the circulation. The insulation of brain from changes in circulating cholesterol is achieved by the blood–brain barrier (BBB). The highest rate of cholesterol synthesis in the CNS occurs during early stages of development especially when myelination is occurring (Jurevics and Morell, 1995, 1997). The mature brain continues to synthesize cholesterol but at lower levels (Mcmillan et al., 1972; Boyles et al., 1985). The finding that the half-life of cholesterol in the adult human brain is approximately 5 years, indicates that it must have an efficient cholesterol recycling mechanism (Bjorkhem et al., 1998).

**FIGURE 1:**
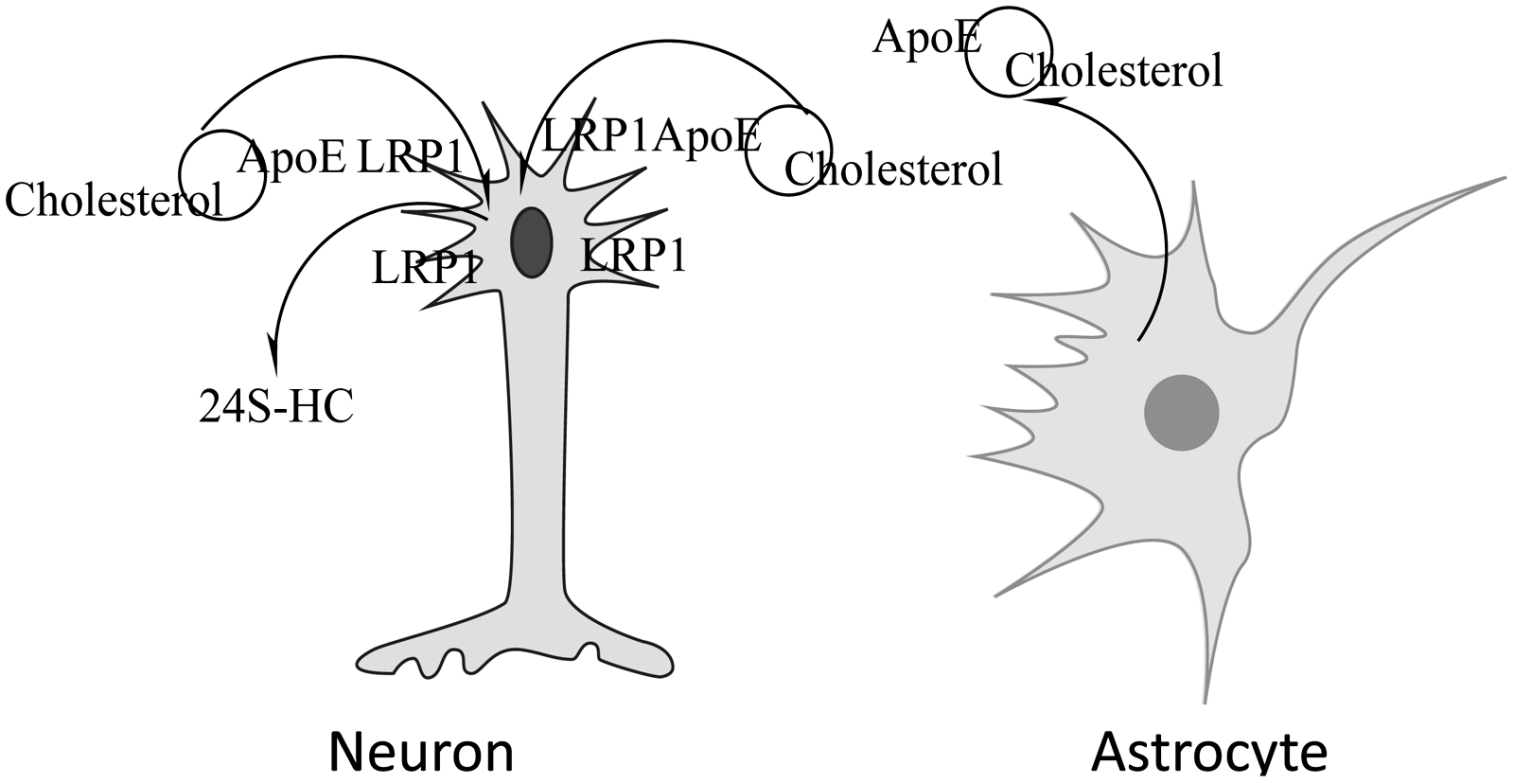
**Neuronal acquisition/loss of cholesterol.** Cholesterol synthesized by astrocytes is packaged in lipoproteins containing ApoE, released from the cell and taken up by neurons via the lipoprotein receptor-related protein 1 (LRP1)-mediated endocytosis. Cholesterol can be removed by conversion to 24S-hydroxycholesterol, a compound that can cross the BBB, by the action of cholesterol 24-hydroxylase.

In the brain, oligodendrocytes, astrocytes and neurons are capable of synthesizing cholesterol (Saito et al., 1987; Suzuki et al., 2007). Most of the cholesterol synthesized by oligodendrocytes is found in myelin. Neurons have a high need for cholesterol due to membrane turnover during synaptic transmission. Instead of synthesizing their own, neurons obtain most of their cholesterol from astrocytes (Srivastava et al., 1997; Stone et al., 1997) which when packaged with ApoE, also synthesized by astrocytes (Pitas et al., 1987a,b; Schmechel, 1993; LaDu, 1998; Dolev and Michaelson, 2004), is delivered to neurons where it is bound by cell surface lipoprotein receptors such as low density lipoprotein receptor (LDLR) and LDLR- related protein 1 (LRP1) that recognize ApoE prior to endocytosis. Though other apolipoproteins are also found in the CNS, i.e., Apo AI, ApoJ, ApoD, evidence suggests that ApoE is highly abundant in the brain and is responsible for the shuttling of cholesterol between astrocytes and neurons (Bjorkhem and Meaney, 2004). A schematic depicting cholesterol metabolism in the CNS is shown in **Figure 1**.

In addition to recycling of cholesterol, there is need for removal of excess cholesterol from the brain. Due to its hydrophobic nature, cholesterol cannot cross the BBB. Two mechanisms have been proposed for its removal; cholesterol hydroxylation and ApoE-dependent eﬄux. The presence of ApoE-bound cholesterol in the CSF indicates that some excretion of brain cholesterol occurs this way but the mechanism is not understood. Based on the rate of CSF renewal and the amount of cholesterol found in the CSF, it has been estimated that 1–2 mg cholesterol may be eliminated from the brain via the CSF each day (Pitas et al., 1987a). The major mechanism of cholesterol clearance from the brain is its conversion by the enzyme cholesterol 24-hydroxylase (CYP46A1) to 24S-hydroxycholesterol (24S-HC), a metabolite that can traverse the BBB (Lutjohann et al., 1996; Bjorkhem et al., 1997, 1998). Introduction of the hydroxyl group in the side chain of the cholesterol molecule causes a rearrangement of membrane phospholipids that allows excretion of the oxysterol in an energetically favorable manner (Kessel et al., 2001). The direction of movement is mediated by the concentration gradient (Bjorkhem and Meaney, 2004). Because almost all of the 24S-HC originates from the brain, it has been suggested that it can be used as a marker of brain cholesterol homeostasis. Consistent with this view, a number of studies have shown altered levels of 24S-HC in the CSF or plasma of patients with neurological diseases such as AD and multiple sclerosis (MS; Bretillon et al., 2000a; Lutjohann et al., 2000; Leoni et al., 2002). These studies also highlight the importance of a normal flux of cholesterol across the CNS that for healthy humans is approximately 0.09 mg/day per kg (Saito et al., 1987) and 1.4 mg/day per kg in mice (Panzenboeck et al., 2002). In people with AD cholesterol eﬄux across the CNS is elevated and is proportional to the severity of dementia (Pfrieger, 2003a). A similar trend was observed in a mouse model of Niemann Pick C1 disease (NPC1), where the increase in cholesterol eﬄux from the brain was associated with increased neurodegeneration (Panzenboeck et al., 2002).

### ROLE OF CHOLESTEROL IN THE BRAIN

Two major functions for cholesterol in the nervous system are in myelination and synaptic transmission. In addition, it is an essential constituent of cellular membranes and of detergent insoluble lipid rafts, areas enriched in sphingolipids and proteins involved in signal transduction, where it can influence signal transduction and cellular processes. The importance of cholesterol in the brain is evident from the fact that it has ∼25% of the body’s cholesterol (Bjorkhem and Meaney, 2004). In the brain, cholesterol is enriched in myelin (i.e., oligodendroglia) and is an essential component of membranes of all cells. Notably, myelin consists of ∼70% lipids [mostly cholesterol, phospholipids, and glycosphingolipids (GSLs), i.e., galactocerebroside in molar ratios of ∼4:4:2] and 30% proteins (Bjorkhem and Meaney, 2004). The high lipid content of myelin is consistent with the membrane properties required for its role in supporting saltatory conduction. Myelin is produced by oligodendrocytes (Miller, 2002) and cholesterol enrichment in myelin reduces permeability to ions allowing the action potential to move down the axon without diffusing across the membrane (Bjorkhem and Meaney, 2004). It has been shown that a high cholesterol content is needed for proper myelination (Saher et al., 2005). A deficiency in oligodendrocyte cholesterol synthesis was shown to delay proper myelination (Marcus and Popko, 2002; Saher et al., 2005) and to be crucial for the development and maintenance of myelin membranes (Colognato et al., 2002; Gielen et al., 2006; Debruin and Harauz, 2007). Low cholesterol levels in an aging brain could contribute to the loss of myelination that Bartzokis (2011) postulated was an underlying cause of multiple degenerative brain disorders.

Cholesterol content of a membrane regulates its fluidity with greater amounts of cholesterol making them more rigid and lesser amounts making them more fluid and allowing more permeability to ions (Barenholz, 2002). Therefore, disruption of cholesterol distribution in the membrane can affect its function in maintaining normal cell activity. Consistent with this view, altered dendritic morphology was observed upon depletion of membrane cholesterol in cultured neurons (Holtzman et al., 1995). Disruption of lipid homeostasis significantly alters CNS structure and function possibly by affecting the composition of lipid rafts found in neurons, astrocytes, and oligodendrocytes (Tsui-Pierchala et al., 2002; Gielen et al., 2006; Debruin and Harauz, 2007). Changes in cholesterol concentration can also affect cell surface availability of the carbohydrate moieties of GSLs (Novak et al., 2013) which in turn might affect a cell’s ability to respond to GSL-carbohydrate binding proteins.

Several studies have shown that cholesterol is needed for endo- and exocytosis and plays a crucial role in synapse structure and function (Pfrieger, 2003b; Takamori et al., 2006). Consistent with this, cholesterol is enriched in presynaptic terminals and its pharmacological depletion reduces synaptic transmission (Suzuki et al., 2004). Several key synaptic proteins such as synaptophysin and the soluble NSF attachment protein receptor (SNARE) proteins are either predominantly found in lipid rafts or must be recruited into them in order to effectively interact with other proteins to promote neurotransmitter release and transmission. These effects are thought to be mediated by direct interaction of cholesterol with synaptophysin (Thiele et al., 2000), and formation of the SNARE complex is cholesterol-dependent (Lang et al., 2001; Mitter et al., 2003). Moreover, Suzuki et al. (2007) showed that an increase in the cholesterol content of lipid rafts, induced by treatment with brain derived neurotropic factor (BDNF), resulted in increased expression of raft-associated presynaptic proteins, changes associated with synapse development. Collectively, these studies highlight the importance of cholesterol in regulating expression of synaptic proteins and for synaptic transmission.

### BRAIN vs. PLASMA CHOLESTEROL IN AD

Findings regarding cholesterol content in the plasma of aging and AD individuals are inconsistent with regards to whether elevated or lower serum cholesterol is a risk factor for AD. A consistent observation in human studies is that high serum cholesterol in mid-life can increase the risk of developing AD later in life, but is associated with improved cognition in elderly individuals (Schreurs, 2010). Similarly, lower total serum cholesterol (Kim et al., 2002) and lower high density lipoprotein (HDL) cholesterol were found to be associated with poor cognition in the elderly (Atzmon et al., 2002). However, status of brain cholesterol could be a better predictor of AD pathogenesis. With regard to the exchangeability between serum and brain cholesterol, the idea that under normal conditions cholesterol from serum does not cross the BBB into the brain is well-established. Support for this concept was provided by studies showing no incorporation of label into lipids of the brain or spinal cord after administration of D_2_O to adult rats (Waelsch et al., 1940). These findings have been replicated by several investigators (Jurevics and Morell, 1995; Dietschy and Turley, 2001). Intravenous injections of ^14^C-labeled cholesterol into healthy volunteers and pregnant women resulted in no label being found in the brains of the healthy volunteers (Meaney et al., 2001) or fetal brain tissue (Plotz et al., 1968), emphasizing that almost all cholesterol in the brain comes from *de novo* synthesis with little to none from plasma under normal physiological conditions. Surprisingly, brain endothelial cells have been shown to take up a small amount of LDL cholesterol via LDL receptors expressed on their luminal surface (Dehouck et al., 1994, 1997). It has been hypothesized that uptake of small amounts of plasma lipoproteins into the CNS could occur at levels too low to be detected by current methods (Dietschy and Turley, 2001). However, significant uptake of cholesterol from plasma could occur under pathological conditions where the integrity of the BBB is compromised or in cases of chronic hypercholesterolemia. This possibility is supported by studies showing a number of metabolic changes in the brains of individuals/experimental animals fed diets high in cholesterol. Currently, knowledge is limited in this respect and further studies are needed to elucidate the effects of dietary cholesterol on brain cholesterol, especially in cases of neurological disease.

### CHOLESTEROL HOMEOSTASIS AND AD PATHOLOGY

There is substantial epidemiological, genetic, and molecular evidence of disruption of cholesterol homeostasis in AD (Shobab et al., 2005; Sjogren et al., 2006). Mutations in several genes involved in cholesterol uptake, such as LRP and the very-low-density lipoprotein (VLDL) receptor (Zerbinatti and Bu, 2005), as well as in enzymes that regulate cholesterol catabolism such as Cyp46A1 (Wolozin, 2003; Vaya and Schipper, 2007), have been associated with increased risk of AD. In addition epidemiological studies revealed increased susceptibility to AD in patients with elevated plasma cholesterol levels in mid-life (Jarvik et al., 1995; Notkola et al., 1998; Roher et al., 1999). However, studies of cholesterol in brains obtained from animals with AD symptoms and AD human brain autopsy tissue have yielded inconclusive results regarding whether high or low brain cholesterol is a risk factor for AD. The lack of agreement between studies could be due to analysis of cholesterol content from different regions/domains, i.e., total brain cholesterol vs. membrane cholesterol vs. cholesterol in lipid raft domains. It has been reported that while the asymmetric distribution of cholesterol in the plasma membrane of aged mice was reduced relative to that of younger mice, total membrane cholesterol was unaltered (Igbavboa et al., 1996). However, cholesterol content of lipid rafts from brains of older mice expressing either human ApoE3 or ApoE4 was greater than in those from brains of younger animals (Igbavboa et al., 2005). It has also been reported that brain cholesterol increases in certain conditions with age, i.e., NPC1 (Vance, 2006) while studies of other conditions provided evidence for a decline in total brain cholesterol, i.e., AD (Ledesma, 2003) and Huntington’s disease (Valenza et al., 2005). In addition to total brain cholesterol, maintenance of cellular cholesterol content is crucial in mediating APP processing via alpha and gamma secretases (Bogdanovic et al., 2002; Cam and Bu, 2006) and therefore has physiological relevance to AD. Ginsberg et al. (1993a,b) have shown that in AD brains cell membranes are less stable due to altered lipid composition. In sum, experimental studies have shown that either abnormally high or low cholesterol can be associated with pathogenic manifestations seen in neurological diseases. Excessive loss of brain cholesterol would be expected to be particularly devastating because the rate of cholesterol synthesis in the brain decreases with age and under normal conditions there is no influx of cholesterol from the plasma into the brain.

ApoE is a major apolipoprotein and cholesterol carrier in the brain (Mahley, 1988). It is synthesized and secreted primarily from astrocytes as part of small dense cholesterol containing lipoproteins (Pitas et al., 1987a,b; Schmechel, 1993; LaDu, 1998; Dolev and Michaelson, 2004). Neurons take up astrocyte-released ApoE via LRP1 mediated endocytosis (Herz, 1988; Jeon and Blacklow, 2005). In humans, ApoE exists as three isoforms (E2, E3, E4; Mahley, 1988). The association of ApoE4 as a strong risk factor for AD has been well established (Mahley, 1988; Corder, 1993; Mahley et al., 2006). Despite advances in understanding how ApoE functions, the molecular mechanisms by which ApoE4 contributes to AD are not completely understood. In the general population, ApoE3 is the most common isoform (allele frequency 77–78%), followed by ApoE4 (15%; Mahley, 1988). However, ApoE4 is present in ∼40% of AD patients (Corder, 1993). Isoform-specific effects in cholesterol transport were observed with ApoE4 being less efficient than ApoE3 (Michikawa et al., 2000; Gong, 2002; Rapp et al., 2006). There is also evidence that ApoE3-expressing astrocytes can supply more cholesterol to neurons than those expressing ApoE4 (Gong, 2002). Recent reports about the ability of neurons to recycle ApoE and retain ApoE inside cells (Fazio et al., 2000) have led to the hypothesis that ApoE may play additional roles in mediating cell signaling and intracellular-homeostasis. One proposed role, that of an antioxidant, is based on its ability to bind metals such as copper, iron, and zinc, with its highest binding affinity being for iron (Miyata and Smith, 1996). Interestingly, the antioxidant and metal binding activities of ApoE were found to be allele-specific, with ApoE4 less efficient in binding metals (Mutter et al., 2004) and reducing oxidative stress than ApoE3 (Miyata and Smith, 1996).

### SPHINGOLIPIDS IN NEURODEGENERATIVE DISEASES

Sphingolipids contain sphingosine as their basic building block and are enriched in lipid rafts. When substituted with a fatty acid in an amide linkage on C2, ceramide is produced that when linked to phosphorylcholine yields sphingomyelin and when glycosylated yields a GSL. Sphingolipids function in cell–cell recognition, signaling cascades that result in cell proliferation, apoptosis, stress responses, inflammation, differentiation, and axon growth (Venable et al., 1995; Hannun and Obeid, 2002; Spiegel and Milstien, 2002; Lavieu et al., 2006; Snider et al., 2010). Unequivocal evidence for the need for appropriate synthesis of sialylated GSLs was provided by the finding that brains of children who lacked the ability to synthesize GM3 failed to develop normally (Simpson et al., 2004). Dysregulation of GSL metabolism has also been implicated in a number of metabolic and neurological diseases such as Fabry disease, Krabbe disease, Gaucher disease, Tay-Sachs disease, Metachromatic leukodystrophy, Niemann-Pick disease, AD (Haughey et al., 2010; He et al., 2010; Ryland et al., 2011).

Several studies have shown abnormalities in the lipid content and expression of enzymes regulating their content in AD brains. For example, the total phospholipid and sulfatide content in AD brains was decreased compared to that in controls (Skinner et al., 1989; Soderberg et al., 1992; Gottfries et al., 1996; Pettegrew et al., 2001; Han et al., 2002; Cheng et al., 2003). On the other hand, ceramide, a pro-apoptotic lipid was found to be elevated in the brains (Cutler et al., 2004) and CSF of patients with AD (Satoi et al., 2005). Ceramide can be produced by hydrolysis of sphingomyelin via sphingomyelinases, or by *de novo* synthesis from fatty acyl CoA and sphingosine. It has been proposed that oxidative stress and other age-related factors contribute to the age-related accumulation of ceramide and induction of apoptotic signaling in neurons and other cells (Kolesnick and Kronke, 1998; Cutler et al., 2004; Costantini et al., 2005; Perez et al., 2005). He et al. (2010) replicated the findings of a reduction in sphingomyelin and an elevation of ceramide in AD brains made by Satoi et al. (2005). In addition, they also found reduced levels of sphingosine-1-phosphate (S1P), a pro-survival metabolite, which correlated significantly with the levels of Aβ peptide and hyperphosphorylated tau protein (He et al., 2010).

In addition to the sphingolipids mentioned above, the relationship of gangliosides, a sub-class of GSLs, to AD pathogenesis has been investigated. Gangliosides are found in their highest concentration in the gray matter of the brain, with GM1, GD1a, GD1b, and GT1b accounting for 65–85% of them (Schengrund, 2010). Interestingly, total ganglioside content was found to be reduced in most regions of brains from early onset or familial cases of AD, while in cases of late-onset or sporadic AD, ganglioside reduction was observed only in the temporal cortex, hippocampus and frontal white matter (Svennerholm and Gottfries, 1994). These observations suggest an age-dependent and/or region-specific pattern of distribution that could be differentially altered in AD.

Though reduction of total brain ganglioside levels in AD has been reported, significant elevation of certain ganglioside species has also been consistently reported. For example, GM1, one of the major gangliosides in brain that is enriched in lipid rafts, was found to be significantly elevated in brains of AD patients (Svennerholm and Gottfries, 1994) and, in lipid rafts isolated from their frontal and temporal cortices (Molander-Melin et al., 2005) A similar observation was made in a neuroblastoma cells expressing the H63D-HFE variant (Ali-Rahmani et al., 2014a) suggesting these changes are driven by HFE genotype and that a model for determining the mechanism for HFE impact on lipid rafts exists

Additional support for the role of gangliosides in AD was provided by the observation that GM1 stimulated production of Aβ by increasing γ-secretase activity (Zha et al., 2004), that it binds to Aβ with high affinity (Ariga et al., 2001), and could serve as a “seed” for Aβ aggregation by promoting formation of toxic Aβ fibrils (Choo-Smith et al., 1997; Yanagisawa, 2005; Kimura and Yanagisawa, 2007; Okada et al., 2007; Yamamoto et al., 2007). Indeed, GM1 was found bound to Aβ in amyloid plaques in AD brains (Hayashi et al., 2004). It has also been shown to influence AD pathogenesis by altering calcium homeostasis. It has been shown that plasma membrane-associated GM1 can enhance intracellular Ca^2+^ levels by increasing influx of extracellular Ca^2+^ (Ledeen and Wu, 2002; Wu et al., 2007) which might affect calcium-mediated phosphorylation of tau and APP (Schengrund, 2010). In addition to affecting APP and Ca^2+^ homeostasis, GM1 has been found to be associated with altered content of other lipids such as cholesterol. Molander-Melin et al. (2005) reported that an elevation of GM1 and GM2 in lipid rafts isolated from AD brains was associated with a concomitant decrease in their cholesterol (Molander-Melin et al., 2005). Similarly, pharmacological depletion of cholesterol in murine neuroblastoma cells resulted in a significant elevation in the recognition of GM1 (Petro and Schengrund, 2007), suggesting an inverse relationship between GM1 and cholesterol. The fact that both lipids are present in rafts supports the hypothesis that significant changes in either or both could affect the ability of rafts to modulate a number of cellular processes affecting neuronal function. Collectively, these studies show that sphingolipids play important roles in AD pathogenesis. Further elucidation of factors that can influence sphingolipid composition is needed. Factors inducing oxidative stress such as iron are potential targets. Future investigation in this area will further our understanding of the connection between iron and lipid metabolism and will help in development of better treatment strategies.

### LIPID RAFTS IN AD

Given the abundance of cholesterol and sphingolipids in the brain, lipid rafts have been proposed as critical for regulating processes such as neuronal signaling, neuronal cell adhesion, axon guidance and neurotransmission required for normal brain function. Rafts have been shown to facilitate intramembrane proteolysis to activate or inactivate several transmembrane proteins such as APP and receptors involved in neurotrophin signaling (Landman and Kim, 2004; Vetrivel et al., 2005). In addition, ionotropic receptors, pre-synaptic SNARE complex proteins (i.e., synaptophysin), as well as, post synaptic receptors [such as *N*-methyl-D-aspartic acid (NMDA) and gamma-aminobutyric acid (GABA)], and key proteins involved in myelination (i.e., CNPase, MBP) are known to localize in lipid rafts and modulation of lipid raft composition has been shown to affect their function (Marta et al., 2004; Stetzkowski-Marden et al., 2006; Willmann et al., 2006; Besshoh et al., 2007; Huang et al., 2007). Changes in the composition of rafts by depletion of cholesterol or sphingolipids can affect the structure (assembly and disassembly) and function of lipid rafts and ultimately, cellular phenotype (Keller et al., 2004; Veatch and Keller, 2005). Evidence is also accumulating to support the concept that there is a dynamic equilibrium between cholesterol and sphingolipids within the rafts and that when levels of one of these components is altered it affects the concentration of the other (Ali-Rahmani et al., 2011).

The roles of lipid rafts in the brain have been under intense investigation, particularly in the context of neurodegenerative diseases. Relevant to AD are studies showing loss of lipid rafts in the temporal cortex (Molander-Melin et al., 2005) and reduced cholesterol in rafts isolated from hippocampi of AD patients (Ledesma, 2003; Abad-Rodriguez et al., 2004). These observations are consistent with findings of significant loss of total brain cholesterol in advanced stage AD patients (Abad-Rodriguez et al., 2004). Studies focused on investigating the role of lipid rafts and their cholesterol content in amyloidogenesis have yielded conflicting results. Analyses of the effect of changes in the cholesterol content of lipid rafts, sites where APP and presenilin-1 (enzyme that cleaves APP to generate toxic Aβ) localize or are recruited to in order to promote amyloidogenesis, indicated that a high cholesterol content favored it (Bouillot et al., 1996; Lee et al., 1998; Morishima-Kawashima and Ihara, 1998; Hayashi et al., 2000). In contrast studies in which cholesterol was depleted from neuronal cells expressing human APP resulted in colocalization of presenilin-1 and APP in non-raft domains and increased Aβ production (Abad-Rodriguez et al., 2004). These findings along with the evidence of low total brain cholesterol in AD patients support the hypothesis that therapeutic approaches designed to lower brain cholesterol could be detrimental to normal brain function. In fact, accumulating evidence indicates that loss of cholesterol can promote neurodegeneration as lower serum/total brain/membrane cholesterol have been implicated in increased risk of Parkinson’s (Du et al., 2012), ALS (Albert, 2008; Chio et al., 2009), Smith–Lemli–Opitz syndrome (Jira et al., 2003), Huntington’s (Valenza et al., 2005), Alzheimer’s (Ledesma, 2003; Wellington, 2004; Wolozin, 2004), and Niemann–Pick Type C (Vance, 2006) diseases. In summary, lipid rafts modulate a number of cellular processes that affect neuronal function and alterations in their composition (cholesterol and sphingolipid content) can influence cellular phenotype. Investigation of lipid rafts in the context of iron related disorders such as HH, AD, and iron deficiency anemia are exciting new areas of research which should be further explored to unravel potential signaling pathways underlying iron related disorders that might be treated by modulation of the lipid composition of rafts.

## IS THERE A LINK BETWEEN IRON AND CHOLESTEROL METABOLISM?

Contribution of iron to neurodegeneration in AD has also been proposed to occur based on its ability to influence four pathways implicated in AD: (1) APP metabolism (Mantyh et al., 1993; Bodovitz et al., 1995; Schubert and Chevion, 1995; Rottkamp et al., 2001; Rogers et al., 2002; Kuperstein and Yavin, 2003), (2), the loss of calcium homeostasis (Hidalgo and Nunez, 2007), (3) the degradation of a subset of microglia (Lopes et al., 2008), and (4) oxidative stress (Honda et al., 2004; Liu et al., 2006; Smith, 2006; Castellani et al., 2007, 2012). Here we propose that the effect of iron on lipid metabolism is also a potential contributor to AD.

Despite the efforts that have been made to identify factors that can trigger the pathological events associated with AD the sequence of events is still not clear. Accumulation of Aβ has been thought to be the triggering event, preceding NFT formation, and ultimately culminating in synaptic failure and memory impairment. However, other evidence indicates that tau pathology may precede formation of amyloid plaques. The evidence of memory decline accompanied by symptoms characteristic of AD, in the absence of amyloid plaques and/or NFTs, supports questioning whether these pathological markers are the only causative agents. Since molecular phenotypes such as oxidative stress, synaptic failure, neuronal loss, and cognitive decline, characteristics associated with AD, have been shown to result from disruption of a number of the pathways discussed above, one can easily argue that there is no linear sequence of events that causes AD. A good example of an interconnection between pathways that may have relevance to AD lies at the intersection between iron and cholesterol metabolism. Both iron and cholesterol metabolism have been independently implicated in the etiology of AD as described above. A large number of studies have established that disruption of cholesterol metabolism is associated with multiple aspects of AD, including APP metabolism, Tau phosphorylation, synaptic integrity and transmission, and cognitive function. Iron dyshomeostasis has also been shown to contribute to the above-mentioned aspects of AD, suggesting an interactive cross-talk between the two pathways that may result in synergistic deleterious effects contributing to the development of AD. Evidence is accumulating for a potential link between iron and cholesterol metabolism in the context of atherosclerosis, yet this link and its relevance to AD remains largely unexplored. There is some evidence to support this association. (1) Iron is a required cofactor for a number of enzymes involved in cholesterol metabolism so it is logical to speculate that alteration in iron content could influence the activity of those enzymes. (2) Iron, ApoE, and cholesterol have all been found in association with extracellular amyloid plaques and intracellular neurofibrillary tangles, hallmark features of AD. (3) Iron is a potent source of oxidative radicals that have been consistently reported to affect membrane lipids and thereby influence lipid homeostasis. (4) Rabbits fed a cholesterol-rich diet were found to accumulate iron and Aβ deposits in the brain, and to have increased mortality (Ghribi et al., 2006). Finally, and perhaps most relevant are the recent findings that carriers of both the ApoE4 (cholesterol transporter) allele and H63D-HFE variant (iron accumulation, cholesterol decrease) had increased risk for and 5.5 year earlier onset of AD (Moalem et al., 2000; Sampietro et al., 2001; Combarros et al., 2003; Percy et al., 2008). Collectively, these studies highlight an area of investigation that could lead to a better understanding of the underlying causes for AD and impact the 25% of the Caucasian population that carries at least one H63D allele.

**FIGURE 2:**
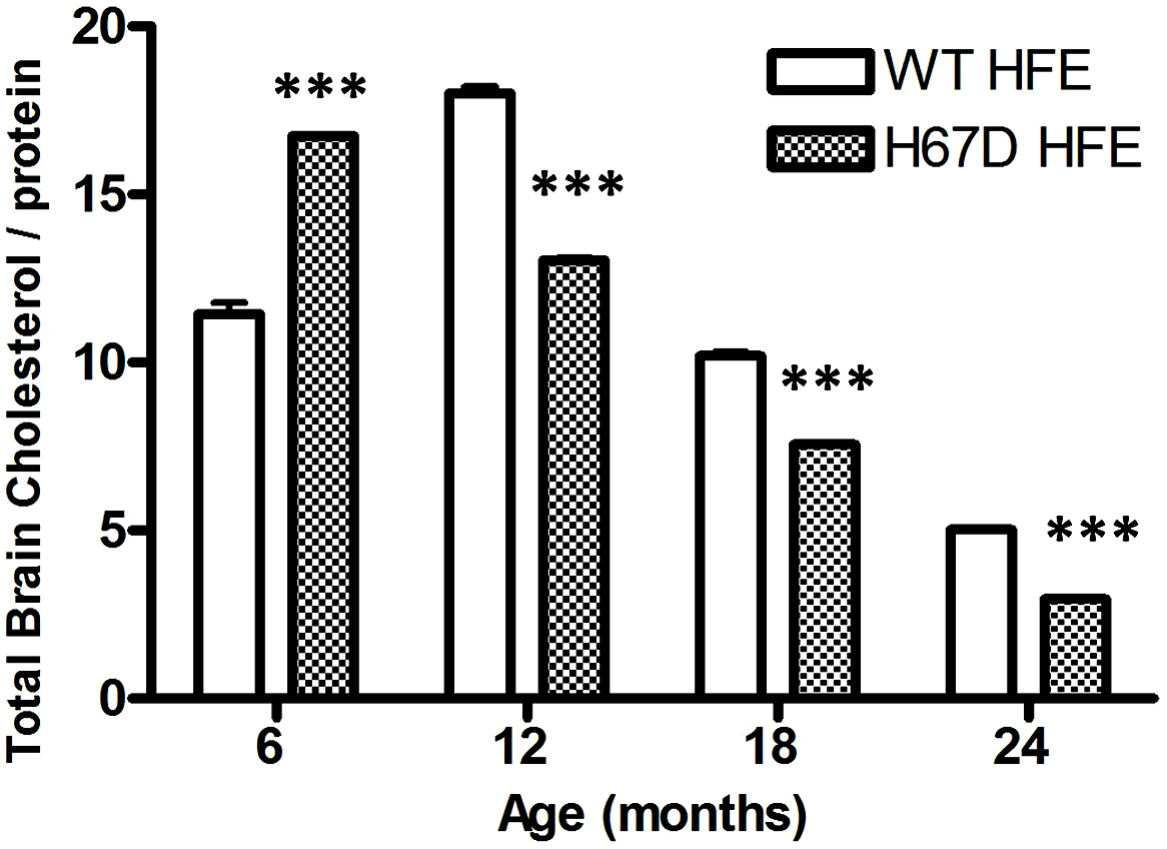
**Total brain cholesterol content of mice expressing WT- or H67D-HFE.** Brains were dissected from mice at 6, 12, 18, and 24 months. After homogenization, protein concentration was determined by Bradford assay (*n* = 7–17 per group including both male and female mice). Lipids were extracted from 100 μl of brain homogenate. Cholesterol content was measured by following the manufacturer’s protocol (Biovision), and is shown as μg of cholesterol per mg of protein (***p < 0.001). Data were analyzed using a two-way ANOVA followed by Bonferroni tests to compare effects of age and genotype on age-related changes in brain cholesterol of HFE variant mice using Graphpad (Prism) software. Error bars represent the standard error of the mean.

The finding that carriers of both the H63D-HFE and ApoE4 alleles have an earlier onset of AD (Percy et al., 2008) indicates that there is a connection between iron, cholesterol, and AD; however, the interaction of iron and cholesterol and its relevance to AD had not been specifically investigated prior to our studies of the effect of H63D-HFE on both (Ali-Rahmani et al., 2014a). While the H63D-HFE-expressing neuroblastoma cells had increased iron content and iron is a required cofactor for a number of enzymes involved in cholesterol synthesis (e.g., HMGCoAR and squalene synthase), the cells expressed less HMGCoAR and had less total cholesterol than WT-HFE-expressing controls. They also had an increased expression of CYP46A1 (Ali-Rahmani et al., 2014a). These observations were recapitulated in studies of the brains of H67D-HFE mice. Brain cholesterol content of H67D-HFE mice was significantly higher in 6 month old mice, however as these mice aged their brain cholesterol content continued to decline much more than in WT-HFE mice (**Figure 2**). Strikingly, a significant negative correlation was found between brain iron and cholesterol content for H67D-HFE mice ages 12–24 months (Ali-Rahmani et al., 2014a). These mice had higher total brain iron content and lower brain cholesterol content than their wild-type littermates. These results are similar to the association found between increased brain (nigrostriatal) iron content and low serum cholesterol in patients with Parkinson’s disease (Du et al., 2012). Though it is believed that cholesterol content of serum is not necessarily reflective of that of brain, it is important to note that in another study it was shown that patients with high serum iron and low serum cholesterol had the highest risk of PD (Powers et al., 2009), indicating the relevance of the interplay between iron and cholesterol metabolism in a neurodegenerative disease.

An inverse relationship between iron and cholesterol content was also observed in several additional studies. For example, Brunet et al. (1997) found that treatment of rats with an iron-salicylate complex resulted in an elevation of iron, products of lipid peroxidation, and reduced total serum cholesterol. Treatment of rats with an iron dextran complex resulted in an elevation of iron and a decrease in total serum cholesterol regardless of whether the rats were fed a normal diet or one high in cholesterol (Turbino-Ribeiro et al., 2003). Similar results were found in earlier studies in which rabbits treated with an iron dextran complex and fed a normal diet were found to have reduced serum cholesterol (Dabbagh et al., 1997). In fact, diet-induced iron overload resulted in reduced expression of liver HMGCoAR and CYP7A1 (Brunet et al., 1999).

**FIGURE 3:**
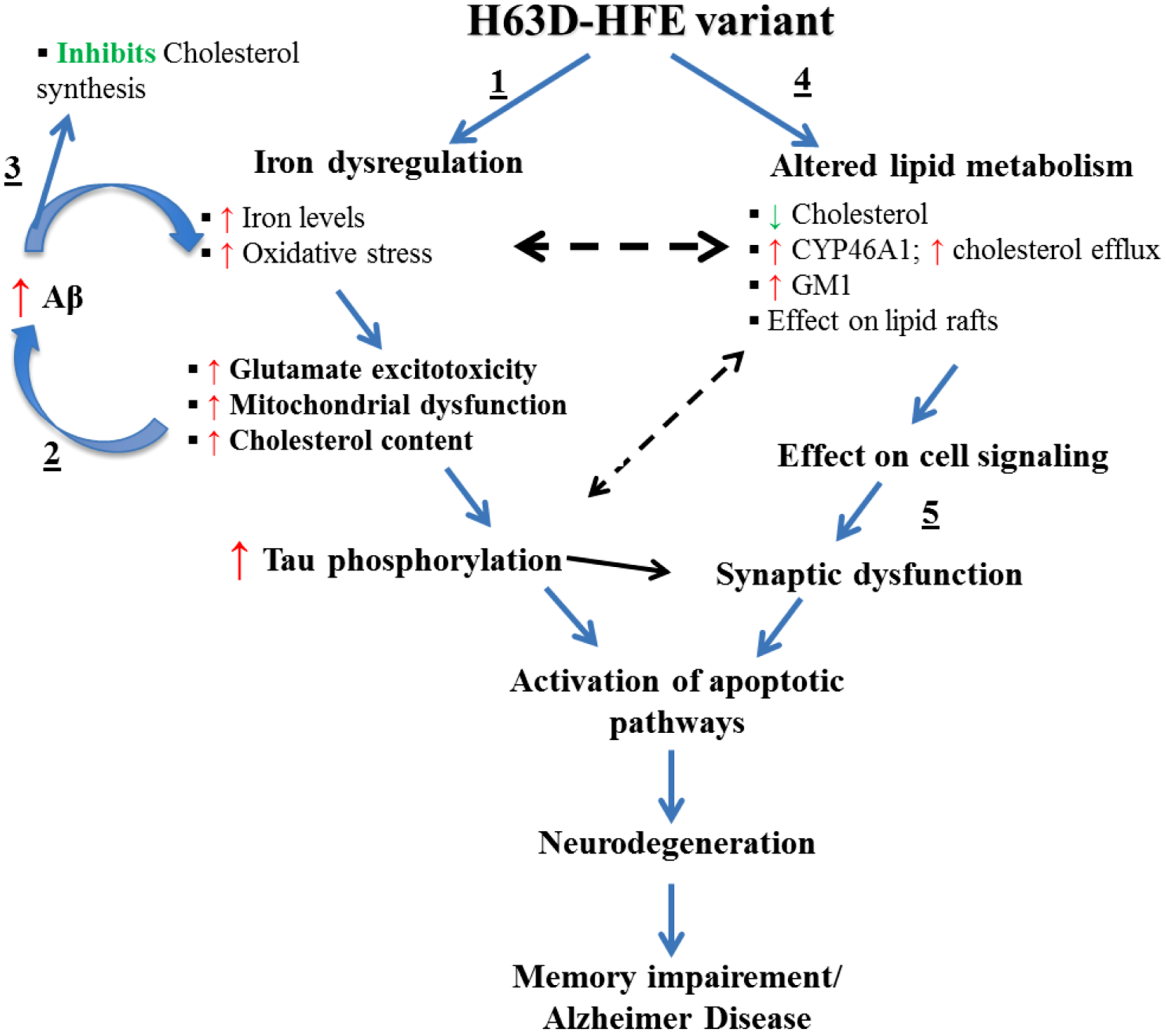
**Effects of the expression H63D-HFE and how they may lead to AD.** We propose that (1) the increase in brain iron induced by the expression of H63D-HFE is initially compensated for by an elevation in brain cholesterol. (2) The elevated iron and cholesterol then synergistically enhance Aβ accumulation and together induce oxidative stress and lipid peroxidation. As the cycle of increased iron, increased Aβ and inhibition of cholesterol synthesis continues, the levels of oxidative radicals supersede the cell’s antioxidant capacity. This affects mitochondrial function, enhances secretion of inflammatory cytokines and other toxic radicals, increases tau phosphorylation, intracellular Ca^2+^, and GM1. (3) As iron accumulation and the concomitant changes it induces occur they start to alter expression of enzymes involved in cholesterol metabolism thereby reducing its concentration. (4) In an effort to survive, cells adapt to a state of low cholesterol content in an effort to limit Aβ production. (5) Over time, due to decreased cholesterol and damage induced by oxidative radicals and other changes, myelin and eventually synapses disintegrate leading to cell death. The resultant neuronal loss would then impair memory function culminating in AD.

One possible explanation for the effects of a high iron diet is that they are mediated by iron-induced oxidative stress (Dabbagh et al., 1994) and lipid peroxidation (Britton et al., 1987). Depletion of energy (ATP and NADPH) due to oxidative stress and lipid-peroxidation mediated membrane damage were shown to cause disruption of lipid synthesis and transport (Kehrer, 1993). These observations coupled with the inverse relationship between iron and cholesterol described above support the hypothesis that the changes induced in iron and cholesterol by expression of H63D-HFE and possibly additional mediators disrupt the normal neuronal function. One possible molecule involved in these interactions is Aβ. The presence of the iron response element (IRE) in the promoter region of the APP gene and the observation of an iron-concentration dependent increase in APP mRNA provides compelling evidence for the role of iron in APP expression and possibly Aβ generation (Bodovitz et al., 1995; Rogers et al., 2002; Rogers and Lahiri, 2004). Interestingly, there is also evidence that Aβ can, in turn, modulate cholesterol and sphingolipid metabolism by regulating the activities of key lipid enzymes (Zinser et al., 2007; Grimm et al., 2012). Specifically, Aβ has been shown to inhibit cholesterol synthesis by inhibiting HMGCoAR (Grimm et al., 2012), suggesting that a feedback mechanism exists between cellular cholesterol and Aβ content. Furthermore, GM1 has been shown to serve as a “seed” for Aβ aggregation into toxic fibrils (Hayashi et al., 2004; Yanagisawa, 2005; Okada et al., 2007). Intriguingly, there is evidence that an increase in GM1, or a change in its accessibility to GM1-binding proteins can occur upon reduction of cellular cholesterol (Lingwood et al., 2011). A link for a relationship between iron, cholesterol, and GM1, was provided by our finding that H63D-HFE human neuroblastoma cells contained more iron (Lee et al., 2007), expressed more cholera toxin binding GM1 (Ali-Rahmani et al., 2011) and had less cholesterol than cells expressing WT-HFE (Ali-Rahmani et al., 2014a). Taken together, our findings and the studies of the role of APP metabolism in AD and its relationship to either iron or cholesterol, provide the basis for the proposed mechanism (**Figure 3**) by which effects induced by expression of H63D-HFE may lead to the symptoms associated with AD.

With the exception of Aβ content, all of the pathological effects indicated in this model have been observed in either cells expressing H63D-HFE or in the brains of mice expressing H67D-HFE. Cellular studies indicated no difference in the levels of total APP or Aβ between WT- and H63D-HFE-expressing cells, but increased sensitivity to Aβ treatment was observed in the latter (Mairuae et al., 2010). Further study of the age-related effects of expression of the H63D variant on APP metabolism in an *in vivo* model should further define the interrelationships of HFE, APP, iron, and cholesterol metabolism, and the mechanisms by which they may contribute to dementia.

As important as it is to study the impact of iron overload on lipid metabolism, the question of what impact high serum and/brain cholesterol levels might have on iron metabolism in the brain should also be investigated. Such a study might help to explain the (1) established link between hypercholesterolemia and atherosclerosis and the fact that they both are significant risk factors for AD, (2) connection between disruption of iron metabolism and atherosclerosis, and (3) impact of a cholesterol enriched diet on iron management. Results from studies in rabbits that were fed a diet high in cholesterol showed an increase in iron accumulation in the brain (Ong and Halliwell, 2004; Ong et al., 2004; Ghribi et al., 2006) and an elevation of lipid peroxidation products (De La Cruz et al., 2000; Gokkusu and Mostafazadeh, 2003). Though passage of dietary cholesterol into the brain is prohibited by the BBB, some metabolites, small molecules, and inflammatory cytokines produced by chronic hypercholesterolemia may be able to cross from the circulation into the BBB and eventually alter the permeability of the BBB to other harmful substances. The observation of iron accumulation in the brain after long-term feeding of a high-cholesterol diet provides support for this argument and raises the question of whether and/or how hypercholesterolemia allows increased passage of iron into the brain parenchyma. The next logical question is then how would individuals with H63D-HFE be affected by these changes? We know that individuals with H63D-HFE have elevated iron levels in liver as well as in the brain (Waheed et al., 1997; Adams et al., 2005; Bartzokis et al., 2010). If these individuals develop hypercholesterolemia, they could have even more passage of iron into the brain, more oxidative stress and other disease-associated consequences. Interestingly, it has been shown that individuals with the ApoE4 allele have high serum cholesterol and an increased incidence of hypercholesterolemia. This observation may provide an explanation for why individuals with both ApoE4 and H63D-HFE have an increased risk and earlier onset of AD. Further elucidation of this mechanism would provide an explanation for why atherosclerosis and hypercholesterolemia, conditions prevalent in many aging individuals, may contribute to the development of AD. In relation to that, studies aimed at teasing out the effects of diet (high-iron or high cholesterol, or both) on mice expressing WT-HFE (have normal iron; able to protect cells from iron overload) should yield results indicating the extent to which diet-induced changes affect iron and cholesterol metabolism in the brain.

### HOW DOES H63D-HFE AFFECT INTERACTIONS BETWEEN GLIAL AND NEURONAL CELLS?

Experimental results indicate that H63D-HFE creates a permissive milieu for pathogenic processes (Lee et al., 2007; Mitchell et al., 2009a,b; Hall et al., 2010, 2011; Mairuae et al., 2010; Ali-Rahmani et al., 2011; Liu et al., 2011). These observations raise the question of how the effects of iron accumulation and resulting oxidative stress, elevation of extracellular glutamate, increased secretion of MCP-1 and 24S-HC in one cell type impacts other cells in the brain and *vice versa*. The importance of this question is highlighted by the characteristic iron-deficient phenotype of macrophages in HH patients, a puzzle that has not yet been solved. It has been shown that although HH patients have a significant iron overload in their livers and other organs, their macrophages appear to be iron-depleted (Cardoso and de Sousa, 2003). Consistent with this observation, cell studies have shown that the normal function of WT-HFE is not only to manage iron uptake but to prevent its eﬄux from cells into the extracellular space (Drakesmith et al., 2002). Because this function is impaired in H63D-HFE cells more iron is released from macrophages (Drakesmith et al., 2002). Considering that microglia in brain function similarly to macrophages in the circulation, expression of H63D-HFE may affect them analogously. If it does, it is likely that H63D-HFE expressing microglia would release more iron than normal and this could affect other cells in the CNS. Support for this is provided by results from a number of studies that examined the effects of iron on cells in the CNS. Zhang et al. (2007) showed that conditioned media from iron-loaded microglia increased survival of oligodendrocytes by delivering iron in ferritin, which could contribute to the proliferative effects on oligodendrocytes. However, lipopolysaccharide (LPS) activation of iron-loaded microglia attenuated the proliferative effects on oligodendrocytes and caused them to release inflammatory cytokines (Zhang et al., 2007). It has also been shown that microglial activation and secretion of inflammatory cytokines can decrease ferritin synthesis via the modulatory effects of nitric oxide on the iron regulatory system (Chenais et al., 2002). It is logical to argue that a decrease in ferritin along with a pre-existing iron-overload and/or increased iron uptake would result in more intracellular free iron that could exacerbate the oxidative damage often seen in neuronal and glial cells in neurological disorders (Rotig et al., 1997; Hirsch and Faucheux, 1998; Smith et al., 1998). So the question is what causes microglial activation? Iron accumulation has been shown to influence glial activation either directly or through induction of other factors such as the MCP-1, a chemokine (Rollins, 1997) shown to cause glial activation (Vrotsos et al., 2009) as well as induction of pro-apoptotic genes and cell death (Zhou et al., 2006). Of particular interest is the finding that cells expressing H63D-HFE have increased secretion of MCP-1 (Mitchell et al., 2009a). Therefore, it is likely that iron accumulation within brain cells affects microglia and this in turn, influences the function of neurons and glia, thereby creating a vicious cycle of events that potentiate stress and damage within the CNS. In such a scenario, endothelial cells, gatekeepers of the BBB, could also be influenced by these changes. In fact, experimental studies have shown that iron accumulation damages endothelial cells by induction of apoptotic pathways (Carlini et al., 2006). Therefore, maintaining iron homeostasis is essential for preserving endothelial function needed to maintain integrity of the BBB. This is crucial for protecting the brain from the harmful effects of chemicals that can be present in the circulation and for maintaining a stable flux of ions into the brain for proper impulse generation and propagation by neurons. The changes in iron and cholesterol induced by expression of H63D-HFE have major effects on the CNS and as such their effects on neuro-glial interactions should be further investigated. For such investigations, the H67D-HFE mouse model could be useful since we found that the animals expressed symptoms associated with human AD (Ali-Rahmani et al., 2014a). Schematic in **Figure 3** summarizes findings from *in vitro* and *in vivo* studies with H63D variant of HFE. The H67D-HFE mouse model could also be used to study the effects of changes in dietary iron or cholesterol, as well as the effects of statins and iron chelators on learning and memory. Results of such studies could provide insights into how gene-environmental interactions may contribute to the pathogenesis of AD.

Because endothelial cells of brain microvasculature come in direct contact with the red blood cells (RBCs) and exchange material such as ions, nutrients, etc., with them, it was postulated that characterization of RBCs may provide useful information about the brain metabolism. The changes in the membranes of RBCs could reflect alterations in the brain and such alterations could be used as biomarkers to diagnose and monitor progression of neurological diseases. In fact, morphology of RBCs and the protein composition of RBC membranes from RBCs isolated from AD subjects were shown to be altered (Sabolovic et al., 1997; Mohanty et al., 2010). Furthermore, oxidative stress associated changes in the phospholipid content of RBC membranes isolated from RBCs from AD patients were observed (Oma et al., 2012) and subsequently a panel of ten plasma phospholipids was shown to predict occurrence of memory impairment in aging individuals (Mapstone et al., 2014). These findings provide support for the hypothesis that RBC lipid dynamics could be important determinants in neurological diseases. Further support for this concept was provided by the observation that RBC membranes from autistic children tended to have reduced cholesterol and increased GM1 relative to those from healthy controls (Schengrund et al., 2012). Based on the finding of similar lipid changes in neuroblastoma cells expressing H63D-HFE, a logical future avenue of investigation would be to compare the lipid and protein composition of RBC membranes from individuals expressing H63D-HFE with those from both healthy people and those with neurological disorders. If indeed a biochemical profile is identified in RBCs from carriers of HFE variants (control, MCI, AD) that is associated with disease pathology, it could be evaluated for its effectiveness as a biomarker to study disease progression. Identification of such a biomarker would significantly aid in future investigations and possibly treatment. Furthermore, the fact that decreased brain cholesterol is associated with disruption of myelin and synapse management as well as cognitive function (Ali-Rahmani et al., 2014a), provides compelling evidence to ascertain whether H63D-HFE is associated with increased risk of developing autism.

### POTENTIAL BIOMARKERS FOR AD PROGRESSION

It is accepted that lipids are crucial for maintenance of normal brain function. However, it is difficult to analyze its lipid content in living individuals. Due to the magnetic properties of iron, brain iron status can be accessed, at least indirectly, by MRI (Bartzokis et al., 1999). However, no such tool is available to monitor the status of brain cholesterol. Owing to the high lipid content of myelin, certain MRI techniques that measure myelination by way of quantifying white matter tracts may provide an idea of lipid status, but no direct conclusions can be made. Because of the difficulty of directly monitoring changes in the brains of living individuals, researchers are searching for potential biomarkers that can be used to identify onset and follow the progression, and response to treatments of neurodegenerative diseases. With respect to brain cholesterol homeostasis, one candidate proposed as an effective biomarker is 24S-HC. Almost all 24S-HC found in plasma and CSF originates from the action of a brain specific enzyme, CYP46A1 which catalyzes the first step in the clearance of cholesterol from the brain (Bjorkhem et al., 1998). Results from a number of studies have shown an elevation of 24S-HC either in the CSF or in the plasma of patients suffering from traumatic brain injury or neurodegenerative diseases (Bretillon et al., 2000b; Cartagena et al., 2010). More specifically, it was found that during the early stages of AD or vascular dementia (Lutjohann et al., 2000) and active periods of MS, high levels of 24S-HC were present in either the CSF or circulation (Leoni et al., 2002), possibly due to ongoing demyelination (Bjorkhem and Meaney, 2004). However, in late stage AD patients’ CSF levels of 24S-HC were found to be reduced (Bretillon et al., 2000a; Leoni et al., 2002, 2006; Papassotiropoulos et al., 2002); they were also reduced in the brains of patients who died from AD (Heverin et al., 2004). The reduction may reflect the extensive neuronal loss seen in late stages of AD. Consistent with these findings, was our observation that expression of the brain-specific enzyme CYP46A1 is elevated in both human neuroblastoma cells expressing H63D-HFE and in the brains of H67D-HFE mice (Ali-Rahmani et al., 2014a). Though the amount of 24S-HC was not measured, the observed concomitant decrease in cellular and total brain cholesterol indicated that it is probable that the expression of H63D/H67D would be associated with higher levels of 24S-HC in serum or CSF or both. It would be useful to confirm this idea using the H67D mouse model. These findings could then provide a rationale for measuring the levels of 24S-HC in the plasma and CSF of individuals carrying H63D-HFE. If a relationship were established between levels of plasma and CSF 24S-HC and incidence or severity of AD, it could be used as a biomarker.

It is also possible that brain volume as determined by MRI could be used as a biomarker for monitoring relative changes in the impact of HFE genotype with normal aging and in disease. This idea is based on the early reports of elevated brain iron in the carriers of H63D (Bartzokis et al., 2010), as well as on reduced volume of brain regions involved in memory in aged H67D-HFE expressing mice, a finding that correlated significantly with the concentrations of brain cholesterol and iron, as well as measures of learning and memory (Ali-Rahmani et al., 2014a). Therefore, these findings suggest that progression of memory decline with normal aging and in patients diagnosed with AD could be monitored by using volumetric MRI measurements. These tools could also be useful for assessing the efficacy of treatment. In summary, the foregoing discussion supports the hypothesis that 24S-HC and brain volume could be useful for monitoring disease progression and possibly treatment efficacy in carriers of H63D-HFE with a neurodegenerative disease.

### IS IT IRON ACCUMULATION OR EXPRESSION OF AN HFE VARIANT THAT IS THE CULPRIT?

H63D and C282Y are the most common variants of HFE and expression of either results in iron overload. Additionally, a number of other cellular processes are also altered in cells expressing these variants. This raises the question of whether the observed changes result from iron accumulation or occur due to a gain-of-function of the mutation. Epidemiological studies indicate that H63D-HFE is a risk factor for AD and C282Y-HFE for certain cancers. Results in **Table 1** summarize previous findings. The fact that both variants of HFE, H63D, and C282Y, are associated with intracellular iron accumulation, yet result in opposing phenotypes, H63D-HFE promoting apoptosis and C282Y-HFE promoting cell survival, indicates that these mutations by themselves could be playing a role independent of their effect on cellular iron status. Supporting this argument are the findings that their subcellular distribution differs. Both WT- and H63D-HFE are found in the plasma membrane, while C282Y-HFE is retained in the ER/golgi. This striking difference is important because the subcellular localization could affect protein-protein interactions and cell signaling. Of potential importance with regard to cell signaling is our observation that while WT-HFE and H63D-HFE were localized in fractions containing lipid rafts, C282Y-HFE was not (Ali-Rahmani et al., 2011). Because lipid rafts provide a platform for a number of cell signaling pathways, an intriguing possibility is that expression of C282Y-HFE alters at least some cell signaling thereby disrupting normal homeostasis. Here it is important to note that although H63D-HFE is present in lipid rafts, its interaction with TfR is altered such that it loses the ability to prevent Tf binding to TfR and iron uptake. Therefore, interactions of both of these mutants with other proteins may be affected. Currently, other binding partners of WT and mutant HFE proteins are not known and should be interrogated in order to elucidate its role in cell signaling. In response to the question of whether the variant itself is a culprit, current evidence indicates that the variant *per se* as well as the resulting iron overload contribute to the observed effects.

**Table 1:** Comparison of *in vivo* and *in vitro* findings of H63D- and C282Y-HFE variants.

	H63D	C282Y	
Iron	↑	↑	Lee et al. (2007)
Oxidative stress	↑	↑	Lee et al. (2007)
Cellular location	Plasma membrane	Endoplasmic reticulum/golgi	Feder et al. (1997)
Cellular cholesterol	↓↓	↑↑	Ali-Rahmani et al. (2014a,b)
GM1	↑↑	↓↓	Ali-Rahmani et al. (2011)
Lipid raft	Yes	No	Ali-Rahmani et al. (2011)
Cellular phenotype	↓Cell proliferation apoptotic	↑Cell proliferation	Lee et al. (2007), Ali-Rahmani et al. (2011), Liu et al. (2011)
Disease risk	AD	Cancer; protective for AD	Moalem et al. (2000), Sampietro et al. (2001), Combarros et al. (2003), Pulliam et al. (2003), Connor and Lee (2006), Liu et al. (2013)

Another potential area for future investigation is to identify proteins that act as molecular switches in the cell and whose expression and/or activity are directly regulated by cellular iron content. This would help to dissect the molecular mechanism(s) underlying a wide array of processes and diseases such as AD and cancer in which iron is implicated. One possible candidate is peptidyl-prolyl cis-trans isomerase (Pin1). It is known to play a key role in regulation of signal transduction pathways (Wulf et al., 2005), particularly in cell proliferation (Lu et al., 1996). Phosphorylation of Pin1 causes it to become inactive. Pin1 modulates the folding, activity, and stability of target proteins by causing their isomerization. The findings that Pin1 is downregulated in degenerating neurons from AD patients (Liou et al., 2003) but its expression and activity is elevated in many cancers including those of the breast, prostate, brain, lung, and colon (Ryo et al., 2001; Wulf et al., 2001; Ayala et al., 2003; Bao et al., 2004), provides support for the argument that Pin1 can serve as a molecular switch. Interestingly, varying concentrations of iron were shown to alter Pin1 phosphorylation and activity in WT- and H63D-HFE cells, indicating that iron can modulate Pin1 activity, but the change in expression is HFE genotype dependent (Hall et al., 2010); presenting another example were HFE genotype could impact cellular basis of disease.

### THERAPEUTIC IMPLICATIONS: IS STATIN THERAPY APPROPRIATE FOR PATIENTS WITH AD AND THE HFE-H63D GENE VARIANT?

Because AD is a multifactorial disease with evidence for the involvement of environmental factors, multiple pathways and genetic mutations in genes with diverse functions, it would be beneficial to identify subpopulations responsive to specific treatments based on their genotype. Identification of genetic and environmental factors, knowledge about their interactions, and resulting pathological markers should allow us to improve therapeutic outcomes. The studies described regarding the effects of a mutation affecting iron uptake on cholesterol metabolism and their relationship to neurodegeneration support our position that eventually medical interventions must take HFE genotype into consideration. For example, the observation that cells carrying the H63D-HFE allele have lower baseline levels of total cholesterol and exhibit slower growth relative to those expressing WT-HFE (Ali-Rahmani et al., 2014a) supports the idea that H63D-HFE positive patients with AD will differ from those with WT-HFE in their response to statin therapy. Our findings that treatment of H63D-HFE cells with a statin that can cross the BBB resulted in decreased cell survival and that statin treatment of the ALS mouse model that also carries the HFE-H63D gene variant worsens the disease (unpublished data) supports this proposal. These data imply that lowering CNS cholesterol could be deleterious to neuronal function, and more so in the carriers of H63D-HFE. Further support for this proposal is provided by a recent clinical trial that showed a reduction in right hippocampal volume after 1 year of atorvastatin therapy in AD patients (Sparks et al., 2008). Though in this study they didn’t stratify based on HFE genotype, such an investigation will be valuable. This finding suggests that in addition to lowering plasma cholesterol levels in AD patients, statin treatment may have reduced brain cholesterol possibly resulting in neuronal loss and atrophy of certain brain regions. Thus, our data are suggesting that use of statins that can cross the BBB, particularly in the presence of HFE gene variants should be evaluated clinically.

In conclusion, the research discussed points out a connection between iron, cholesterol, and neuronal function. This review also provides a synopsis of some of the many questions about the roles that iron, cholesterol, and sphingolipids may have in the evolution of dementing diseases in the brain and introduces a new concept, namely that HFE gene variants dramatically influence cholesterol metabolism.

## Conflict of Interest Statement

The authors declare that the research was conducted in the absence of any commercial or financial relationships that could be construed as a potential conflict of interest.
